# Clinical characteristics of patients with diabetic ketoacidosis at the Intensive Care Unit of a University Hospital

**DOI:** 10.12669/pjms.316.7550

**Published:** 2015

**Authors:** Faiza Qari

**Affiliations:** 1 Faiza Qari, Professor of Medicine, King Abdulaziz University, Faculty of Medicine, P.O. Box 80215, Jeddah 21589, Kingdom of Saudi Arabia

**Keywords:** Diabetic ketoacidosis, Type1 diabetes, Intensive care unit, Saudi Arabia

## Abstract

**Objectives:**

The aims of this study were assessing the frequency of clinical characteristics of patients with severe Diabetic ketoacidosis (DKA) who were admitted to the intensive care unit (ICU) and investigating the relationship between paraclinical (glucose, anion gap, and serum bicarbonate) and clinical parameters in patients with severe DKA.

**Method:**

A retrospective chart review of all adult patients with DKA who were admitted to the ICU at King Abdulaziz University Hospital, Jeddah, Saudi Arabia between January 2012 and December 2013. The data collected include the demographic data, clinical presentation, precipitating factors, duration of hospital stay and mortality rate. The data were analyzed using STAT software.

**Results:**

A total of 60 patients were included. Of these, 50 were men (83.3%). The median age was 23 years (ranging 18-29 years). Newly diagnosed diabetics accounted for 15 (25%) of the cases; the remainder were previously known patients of type1 diabetes on treatment. The main precipitating factors of DKA were insulin treatment cessation (87.5%) and infection/sepsis (39.6%). Serum blood glucose, serum bicarbonate level, and the calculated anion gap did not significantly correlate with clinical parameters of severe DKA.

**Conclusion:**

Most patients with severe DKA who were admitted to the ICU of our institution presented with gastrointestinal symptoms. Non-compliance to insulin therapy was the main precipitating factor of DKA.

## INTRODUCTION

Diabetic ketoacidosis (DKA) is the most common acute hyperglycemic complication of diabetes. According to a recent report,^1^ DKA affects approximately 8 per 1000 diabetics annually. It is associated with significant morbidity and mortality,^2^ with a worldwide mortality rate of 2-10%.^3^

Diabetic ketoacidosis is defined by the triad: hyperglycemia, acidosis and ketonuria. It is the most severe acute metabolic complication of diabetes mellitus.^4^ Current diagnostic criteria and classification of DKA are based on measurements of plasma glucose, arterial pH, serum bicarbonate, anion gap, and mental status alterations.^5^ Most cases of DKA are triggered by cessation of insulin and by infection, especially in developing countries.^6^

Mortality rate is lower in patients that received appropriate treatment: administration of insulin, correction of hypovolumia and electrolytes, and management of precipitating factors.^7^

The aims of this study were to describe the clinical characteristics of patients with severe DKA who were admitted to the Intensive Care Unit at King Abdulaziz University Hospital and to investigate the relationship between paraclinical (glucose, anion gap, and serum bicarbonate) and clinical parameters in patients with severe DKA.

## METHODS

This was a retrospective chart review of the medical records of patients with a diagnosis of DKA who were admitted to the ICU of King Abdulaziz University between January 1, 2012 and December 31, 2013. The inclusion criteria were critically ill patient, above 13 years of age, with a confirmed diagnosis of DKA. Diagnosis of DKA was based on latest criteria namely blood glucose concentration > 11 mmol/L, blood pH <7.3, and serum bicarbonate level < 15 mmol/L.^4^ The decision to admit patients with DKA to the ICU was made by the emergency department physician or internist in charge. Patients with a hyperosmolar hyperglycemic state were excluded from the study. The study was approved by the university’s ethics committee.

### Data collection

The information extracted included from patients’ records demographic data, clinical presentation such as fever, abdominal pain, vomiting, dyspnea, altered sensorium and hypotension; as well as precipitating factors, such as cessation of insulin and sepsis, Sepsis being defined according to the Society of Critical Care Medicine / European Society of Intensive Care Medicine / American College of Chest Physicians consensus conference definitions. The length of hospital stay and mortality were also documented mechanical ventilation was accounted for.

### Identification of laboratory and clinical parameters to determine the severity of diabetic ketoacidosis

We considered plasma glucose, serum bicarbonate levels and anion gap as indicators of the severity of DKA. The anion gap was calculated using the following formula: [(Na^+^) – (Cl^-^ + HCO3- (mEq/L)].

We also determined the severity of DKA based on the patient’s level of consciousness (drowsy, stupor or coma) and the need for mechanical ventilation.

### Statistical analysis

Proportions for dichotomous variables and median (with interquartile range) for continuous variables were calculated to describe patients’ characteristics. Associations between different indicators of DKA (explanatory variables) and outcome variables, alterations in sensorium and need for mechanical ventilation were assessed using regression analysis. Multiple logistic regressions were performed to control for potential confounding variables (age, sex, and presence of infection). To test for statistical significance, the 95% confidence interval was estimated.

All analyses were performed using STAT (StataCorp, College Station, TX, USA) software, version 12. To test for statistical significance, the 95% confidence interval was estimated. Results are expressed as frequency (percent) and mean ± standard deviation.

## RESULTS

### Patient characteristics

Sixty patients were included in the study; aged 18-29 years (median age is 23 years). Of these, 50 (83.3%) were men (Table-I). Newly diagnosed diabetics accounted for 15 (25%) of the cases; the remainder were known cases of type 1 diabetes on insulin treatment. Three patients were on insulin pump therapy. The mean glycated haemoglobin of the sample was 10% (range, 8.6–12.4%).

**Table-I:** Clinical features of patients admitted to the intensive care unit with diabetic ketoacidosis.

Clinical Characteristics	Frequency (percent)
Fever	19 (31.6)
Abdominal pain	50 (83.3)
Vomiting	55 (91.7)
Dyspnea	17 (28.3)
Low blood pressure	8 (13.3)
Altered sensorium	22 (36.6)
Cessation of insulin treatment	52 (86.6)
Infection	23 (38.3)
Need for mechanical ventilation	15 (25.0)

*Abbreviations:* CI, confidence interval.

### Clinical presentations

The most common presentation was gastrointestinal symptoms, including vomiting (n=55; 91.7%) and / or abdominal pain (n=50; 83.3%). Other reported symptoms include dyspnea (n=17; 28.3%), fever (n=19; 31.6%) and hypotension (n=8; 13.3%). Altered sensorium (altered consciousness, disorientation, or stupor / coma) was reported in 22 patients (36.6%). The average length of stay in the ICU was 2.5 days (range, 2-3 days). No mortalities occurred in this sample.

### Precipitating factors of diabetic ketoacidosis

The most common precipitating factor of DKA was insulin cessation (n=52; 86.6%). Infection/sepsis were the second most common precipitating factor (n=23; 38.3%).

### Correlation between laboratory parameters and clinical predictors of diabetic keto-acidosis

Serum blood glucose, serum bicarbonate level, and calculated anion gap were independently correlated with the clinical predictors of severe DKA (Tables II and III).

**Table-II:** Association between indicators of diabetic ketoacidosis and alterations in sensorium among the sample.

Parameters	Crude OR (95% CI)
Blood glucose level	1.00 (0.94 – 1.06)
Serum bicarbonate level	1.02 (0.94 – 1.1)
Calculated anion gab	0.98 (0.93 – 1.03)

* Adjusted for age, sex, and presence of infection. Abbreviations: CI, confidence interval; OR, odds ratio.

**Table-III:** Association between indicators of diabetic ketoacidosis and the need for mechanical ventilation among the sample.

Parameters	Crude OR (95% CI)	Adjusted* OR (95% CI)
Blood glucose level	1.01 (0.94 – 1.08)	1.01 (0.95 – 1.08)
Serum bicarbonate level	1.00 (0.91 – 1.10)	0.99 (0.90 – 1.10)
Calculated anion gab	1.02 (0.96 – 1.08)	1.05 (0.98 – 1.12)

* Adjusted for age, sex, and presence of infection.

Abbreviations: CI, confidence interval; OR, odds ratio.

## DISCUSSION

This retrospective study has described the clinical characteristics of patients with severe DKA who were admitted to the ICU of King Abdulaziz university Hospital in Jeddah. On admission, over four-fifths of our patients presented with gastrointestinal symptoms (vomiting and / or abdominal pain episodes), while one-third presented with altered sensorium (altered consciousness, disorientation, or stupor / coma). These are similar to the reports from other authors that gastrointestinal symptoms and altered sensorium were the commonest presentations of DKA.^8^ However, the rate of gastrointestinal symptoms reported in our study is higher than that reported by these authors. We believe this disparity may be related to differences in sample characteristics. Fifteen patients (25%) of our sample were newly diagnosed type 1 diabetics with DKA as their first presentation, while only 18.2% of the patients in a study by Gavrielatos et al.^8^ were newly diagnosed type 1 diabetics with DKA as a first presentation.

The most commonly used diagnostic criteria for DKA are plasma glucose >250 mg/dL (>13.9 mmol/L), arterial pH <7.3, presence of ketonemia and/or ketonuria.^9^,^10^ Alterations in sensorium and the need for mechanical ventilation are indicators of severe DKA, which has been reported in a retrospective matched cohort study.^6^ Altered mental status in our study was considered to be a predictor of DKA severity. Although the mechanisms underlying the cause of altered sensorium in DKA are unclear, it may be due to different causes, including compromised cerebral blood flow, reduced cerebral glucose utilization, hyperosmolality, high blood glucose concentrations, acidosis, or a direct effect of ketone bodies among many other proposed theories and factors.

The need for mechanical ventilation is another predictor of severe DKA in our study. This could be related to sepsis as a precipitating factor. The diagnostic criteria for sepsis were in accordance to the 2003 International Sepsis Definitions Conference,^11^ including altered mental status as a marker of global hypoperfusion. Tissue hypoperfusion is an important factor in the development of multi-organ failure, which is a measure of severity and the need for mechanical ventilation.^12^,^13^

In previous studies, infection was the most commonly reported precipitating factor in DKA. Other reported precipitating factors include discontinuation of or inadequate insulin therapy, cerebrovascular events, cardiovascular events and drugs.^9^ Furthermore, new-onset type 1 diabetes or insulin cessation in established type 1 diabetes typically results in the development of DKA.^10^

In our study, insulin cessation, either deliberately or inadvertently, was the main precipitating factor of DKA. While patients may be non-compliant to treatment for several reasons—financial constraints, non-availability of insulin, or use of alternative treatments—we believe that DKA can be prevented by educating diabetic patients and their family on the importance of optimal insulin therapy, dietary modification, providing psychosocial support, ensuring and monitoring glycemic control. King Abdulaziz university hospital offers free medical care to expatriates and persons with low economic status; this is why many patients who were admitted to the ICU for DKA were unable to afford treatment.

Outcomes for our patients were favorable with no reported deaths in the study group. DKA has a mortality rate of 1-5%.^10^,^14^ demonstrated in previous studies. Mortality in DKA is rarely due to the metabolic complications of acidosis, but is mainly related to the underlying precipitating illness.^9^

Our study has a number of limitations that warrant consideration. First, it has all the limitations inherent in retrospective studies. Second, the relatively small sample size limits the study’s statistical power. Finally, data available on the long-term outcomes of our patients after discharge from the ICU were not available.

## CONCLUSIONS

Overall, most patients with DKA who were admitted to the ICU of our institution presented with gastrointestinal symptoms. Non-compliance to insulin therapy was the main precipitating factor in these patients, suggesting an important gap in health services delivery. We believe that non-compliant patients might benefit from patient-centered strategies that are focused on simplifying access to medical care and education. Consequently, less privileged patients might benefit from a good healthcare system if a multidisciplinary program is created to alleviate the financial and technical burden of treating DKA. This program should also aim to increase compliance to insulin therapy by providing less privileged patients who lack medical insurance or financial stability with the necessary medication, follow up care and education.
